# Valuable Nutrients and Functional Bioactives in Different Parts of Olive (*Olea europaea* L.)—A Review

**DOI:** 10.3390/ijms13033291

**Published:** 2012-03-12

**Authors:** Rahele Ghanbari, Farooq Anwar, Khalid M. Alkharfy, Anwarul-Hassan Gilani, Nazamid Saari

**Affiliations:** 1 Faculty of Food Science and Technology, Universiti Putra Malaysia, 43400 Serdang, Selangor, Malaysia; E-Mail: raheleghanbari@yahoo.com; 2 Department of Chemistry, University of Sargodha, Sargodha-40100, Pakistan; 3 Department of Clinical Pharmacy, College of Pharmacy, King Saud University, Riyadh, Saudi Arabia; E-Mail: alkharfy@ksu.edu.sa; 4 Natural Products Research Division, Department of Biologicaland Biomedical Sciences, Aga Khan University Medical College, Karachi 74800, Pakistan; E-Mail: anwar.gilani@aku.edu

**Keywords:** Mediterranean diet, high-value components, bioactives, phytochemicals, virgin olive oil, medicinal uses, therapeutic potential

## Abstract

The Olive tree (*Olea europaea* L.), a native of the Mediterranean basin and parts of Asia, is now widely cultivated in many other parts of the world for production of olive oil and table olives. Olive is a rich source of valuable nutrients and bioactives of medicinal and therapeutic interest. Olive fruit contains appreciable concentration, 1–3% of fresh pulp weight, of hydrophilic (phenolic acids, phenolic alchohols, flavonoids and secoiridoids) and lipophilic (cresols) phenolic compounds that are known to possess multiple biological activities such as antioxidant, anticarcinogenic, antiinflammatory, antimicrobial, antihypertensive, antidyslipidemic, cardiotonic, laxative, and antiplatelet. Other important compounds present in olive fruit are pectin, organic acids, and pigments. Virgin olive oil (VOO), extracted mechanically from the fruit, is also very popular for its nutritive and health-promoting potential, especially against cardiovascular disorders due to the presence of high levels of monounsaturates and other valuable minor components such as phenolics, phytosterols, tocopherols, carotenoids, chlorophyll and squalene. The cultivar, area of production, harvest time, and the processing techniques employed are some of the factors shown to influence the composition of olive fruit and olive oil. This review focuses comprehensively on the nutrients and high-value bioactives profile as well as medicinal and functional aspects of different parts of olives and its byproducts. Various factors affecting the composition of this food commodity of medicinal value are also discussed.

## 1. Introduction

The Olive (*Olea europaea* L.) is a small tree, which belongs to the family *Oleaceae* and is native to tropical and warm temperate regions of the world. The tree, famous for its fruit, also called the olive, is commercially important in the Mediterranean region as a prime source of olive oil [1]. The tree is typically distributed in the the coastal areas of the eastern Mediterranean Basin, the adjoining coastal areas of southeastern Europe, western Asia and northern Africa as well as northern Iran at the south end of the Caspian Sea. Although olive is now cultivated in several parts of the world, the Medetarrianen region still serves as the major production area accounting for about 98% of the world’s olive cultivation [2].

According to estimates, the cultivation of olive tree dates back more than 7000 years. Archaelogical evidence indicates that olives were grown commercially in Crete as far back as 3000 BC, by the Minoan civilization [3]. Ancient Greek literature reveals uses of olive oil for body health [3]. In the context of religious importance, olive tree and its fruit (olives) are narrated over several times in the Bible, both in the New and Old Testaments [2] as well as in the Quran [2,3]. Olive is praised as a blessed fruit in Chapter 24 Al-Nur (Quran 24:35).

The olive tree has a long history of medicinal and nutritional values. Over the centuries, extracts from olive leaf have been used for promoting health and preservation. For instance, ancient Egyptians used the leaves to mummify Pharaohs. Similarly, they have been valued as a famous folk remedy to treat fever and some tropical diseases such as malaria [4]. Economically, the fruit of olive is an important commodity as it yields nutritious edible oil with potential medicinal functions [5]. Olives are rarely used in their natural form due to severe bitterness; nevertheless, they are consumed in either one of the two forms, namely oil or table olives. Oleuropein is the bitterness-causing chemical component that must be eliminated from olivesto make them palatable [2,4].

Due to rising awareness about the beneficial effects of optimal nutrition and functional foods among todays’s health conscious cosmopolitan societies, the worldwide consumption of olives and olive products has increased significantly, especially in high-income countries such as the United States, Europe, Japan, Canada and Australia, resulting the rapid development of olive-based products [2,6]. The traditional “Mediterranean diet”, in which olive oil is the main dietary fat, is considered to be one of the healthiest because of its strong association with the reduced incidence of cardiovascular diseases and certain cancers [2,4,7,8]. The health benefits of olive oil are mainly ascribed to the presence of high content of monounsaturated fatty acid (MUFAs) and functional bioactives including tocopherols, carotenoids, phospholipids and phenolics, with multiple biological activities [9,10]. Such components also contribute to the unique flavour and taste of olive oil. As with other crops, the composition of olive and olive oil components varies in relation to various factors, namely cultivar, ripeness and harvesting regime, agroclimatic conditions as well as the processing techniques employed [10].

The main objective of the present review is to compile a comprehensive report, covering nutritional, medicinal and functional aspects of different parts of the olive, thus providing detailed information about the valuable nutrients and high-value bioactives of this multipurpose tree. The technological advancements to be applicable for the potential recovery of valuable components from olive processing wastes have also been discussed.

## 2. Distribution, Cultivation and Production of Table Olives and Olive Oil

The task of identifying and classifying different olive varieties is quite challenging [1]. There are around 2500 known varieties of olives, of which 250 are classified as commercial cultivars by the International Olive Oil Council as described in Table 1 [11]. These commercial cultivars are used for the production of either olive oil or table olives or both. The specific use of a given cultivar is determined by its oil content and size. Olive varieties with oil content less than 12% such as Ascolano, Calamata and Manzanillo are almost exclusively used for table olive production, while those withhigher oil yield (*ca*. 20%) such as Hojiblanca, Verdial, Picual, Gemlik, Nychati Kalamonand Arauco are usually preferred for the purpose of olive oil production [2]. The larger fruits (more than 4 g) are mostly favored for table olive consumption. In the case of table olive production, besides the fruit size, several other physical properties of fruits cultivars such as shape, flesh-to-pit ratio, colour and texture are of key importance (Table 2) but some other factors, namely harvesting procedure, type of cultivation (irrigated or non-irrigated system), and ripening cycle areregarded less important [12].

Naturally, olive fruits have a bitter flavor; hence they are typically subjected to fermentation or cured with lye or brine to make them more palatable. There are three important internationally used practices for preparation of table olives [13].

1. Spanish-style (pickled) green olives in brine
2. Californian-style (pickled) black olives in brine
3. Greek-style natural black olives in brine

In the Spanish and Californian-style method, the bitterness of the olive is removed by using food grade sodium hydroxide treatment that caused several changes in some compounds of the fruit; however triglycerides composition remains unaffected by this treatment. After the preliminary brine treatment, the fruits are rinsed to remove alkali and then fermented in brine for several months. In the case of Greek-style, the fruits are placed directly in the brine to remove oleuropein completely or partially. In the Spanish-style, a lactic fermentation is principally used for green olive brine, while for the Greek-style; natural black olives in brine are fermented mostly using yeasts. In the Californian-style, black olives do not necessarily require fermentation at all. They are treated directly with lye and oxidized, then washed, placed in brine, and packed in cans with heat-sterilization [12].

Olives are known as one of the widely cultivated fruit crops worldover [14]. Presently, approximately more than 750 million olive trees are cultivated worldwide. About 98% of the total surface area, 99% of productive trees and 99% of total olive production belong to the countries around the Mediterranean basin and in the Middle-East. According to an estimate of Food and Agriculture Organization (FAO), in 2009, 9.9 million hectares (ha) were planted with olive trees. Spain, with a total cultivated area of 2,500,000 ha, is the biggest producer, followed by Italy (1,159,000 ha) and Greece (765,000 ha) [15]. Each olive tree produces an average of 15 to 50 kg of olives; depending on the nature of olive and environmental conditions [11]. World olive oil production in 2008–2009 was 2.9 million tones, of which Spain, contributing over 40%, was the top producer. After Spain and Italy, Greece holds third position in world olive oil production, producing approximately 332,000 tons of olive oil annually, of which 82% is extra-virgin olive oil. About half of the annual Greek olive oil produced is exported, but only some 5% of this reflects the origin of the bottled product [15]. Based on the data from the FAO, ten main olive producing countries are located in the Mediterranean region, which produce 95% of the world’s olives as shown in Table 3.

Virgin olive oil (VOO) is obtained solely through physical means by mechanical or direct pressing of the olives under mild thermal conditions that do not lead to alterations in the oil composition. Virgin olive oil is not subjected to any treatment except washing, decantation, centrifugation and filtration [16]. In this regard, the oils produced by solvent extraction or re-esterification processes, and those blended or mixed with other vegetable oils are excluded from the category of VOO.

World olive oil production in 2009 was 2.9 million tones, of which Spain supplied 1/3 of the world’s olive oil followed by Italy (1/4), and Greece (1/5). Greece has the maximum olive oil consumption per capita worldwide (around 23.7 liters/person/year), followed by Spain and Italy with around 13.62 liters and 12.35 liters, respectively (Table 4). On the commercial product value basis, olive oil contributes 15 percent share of the world oil trade [17]. The price of olive oil is usually 2–5 fold higher than that of other vegetable oils depending on the origin, category/type of the oil, and the harvesting period of the olive fruits [17].

## 3. Composition of High-Value Nutrients and Functional Bioactives in Different Parts of Olive

Different parts of the olive are valued for their nutrients and functional food components and health-promoting bioactives.

### 3.1. Olive Fruit

The olive fruit is an oval-shaped drupe and possesses a typical size of 2–3 cm (width and length) and pulp per stone ratios of 3.0–6.5. The olive fruit is essentially made up of 3 parts, epicarp or skin, mesocarp or pulp and endocarp or stone. The epicarp (skin) is covered with wax; during the growth phase the skin colour turns from light green to purple and brown or black. The mesocarp, with a soft, pulpy flesh, accounts for 84–90% (of the total fruit mass) while the hard endocarp (stone) containing the seed or kernel may differ from 13 to 30% of fruit weight. The seed contains 2–4 g oil /100 g. Olive fruit weight may range from 2–12 g, although some varieties may weigh as much as 20 g [18,19].

The growth and ripening of olive fruit is a long process, which takes about 5 months in usual climatic conditions. However, in cold climatic conditions, growth is slower. Olive fruit’s average composition includes water (50%), protein (1.6%), oil (22%), carbohydrate (19.1%), cellulose (5.8%), inorganic substances (1.5%) and phenolic compounds (1–3%). Other important compounds present in olive fruit are pectin, organic acids, and pigments [1]. Organic acids show metabolic activity and are intermediate products resulting from formation and degradation of other compounds [20].

The distribution and structure of the chemical constituents of olive fruit is complex and dependent on parameters including variety, cultivation practices, geographical origin, and the level of maturation. The olive phenols impart antimicrobial properties to different parts of the plant and are also responsible for the extent of browning in the fruit. These phenolic components also contribute towards the sensory and aromatic characteristics of the olive as well as impart pharmaceutical and physiological benefits [1,9,21].

**Figure 1:**
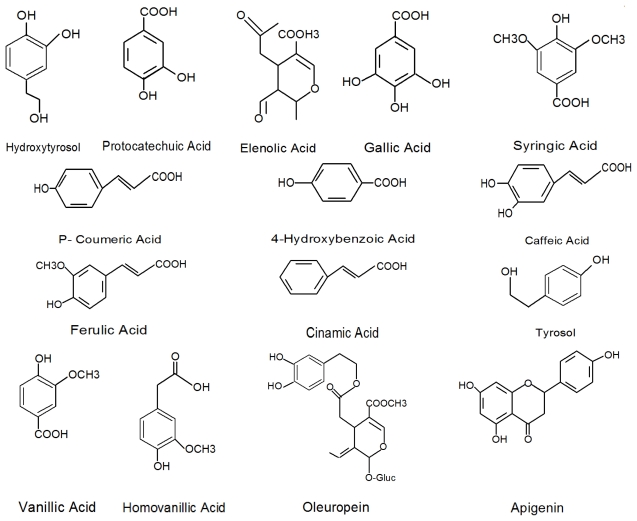

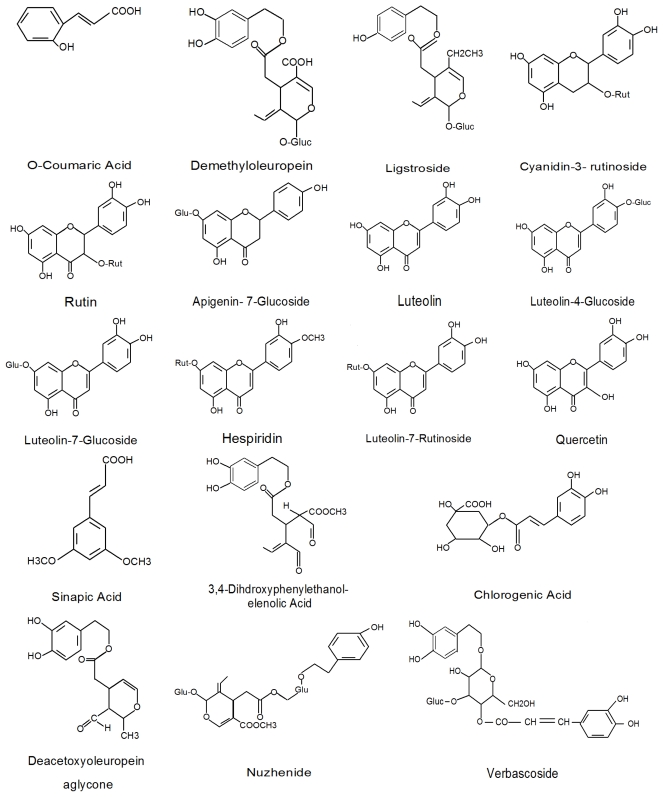
Chemical structure of important bioactives in olive/olive oil [2,28].

Both lipophilic and hydrophilic phenolics are distributed in olive fruit. The main lipophilic phenols are cresols while the major hydrophilic phenols include phenolic acids, phenolic alchohols, flavonoids and secoiridoids; they are present in almost all parts of the plant but their nature and concentration varies greatly between the tissues [1,9]. Phenolic acids are named as secondary aromatic plant metabolites that are commonly distributed throughout the plant kingdom [22,23]. Phenolic acids with basic skeleton of C_6_–C_1_ (hydroxybenzoic acid) such as vanillic acid, syringic acid, gallic acid; C_6_–C_3_ (hydroxycinnamic acid) such as caffeic acid, ferulic acid and sinapic acid, and flavonoids with the chemical structure of C_6_–C_3_–C_6_ such as cyanidin have been investigated in olive fruit [2,4,12,24,25]. The main hydroxycinnamic acid derivative reported in olive fruit is verbascoside [26]; its chemical structure was identified by Andary *et al.* [27] and confirmed by Servili *et al.* [26], as shown in Figure 1.

The main phenolic alcohols of olives include oleuropein β-(3,4-dihydroxyphenylethanol) or hydroxytyrosol and *p*-hydroxyphenylethanol (tyrosol) [2,29]. Flavonoid compounds in olive are mainly comprised of flavonol glycosides such as luteolin 7-*O*-glucoside, rutin, apigenin 7-*O*-glucoside, anthocyanins, cyanidin 3-*O*-glucoside and cyanidin 3-*O*-rutinoside [30,31]. Hydroxytyrosol and tyrosol are present at the highest contents of 76.73 and 19.48 mg/100 g olives, respectively, in comparison to the rest of the phenolic compounds [24]. During ripening of olives, oleuropein is completely degraded, and is almost undetectable when the fruit darkens, but hydroxytyrosol, tyrosol, and verbascoside increase [4]. The presence of such medicinally important bioactive compounds in olive fruit supports the functional foods potential of the major products, table olives and virgin olive oil, produced from this species [1,2,10,12,24,25]. Besides, two important pentacyclic triterpenes, namely oleanolic and maslinic acids with potential antiproliferative activity, have been reported from olive fruit skin [32].

Oleuropein is the major secoiroidoids constituent of unripe olive fruit. The concentration of this compound decreases with maturation, while demethyloleuropein and the dialdehydic form of elenolic acid linked to β-(3,4-dihydroxyphenyl) ethanol), (3,4-DHPEA or hydroxytyrosol) increases [33]. Ragazzi *et al.* [34] was the first to separate and characterize demethyloleuropein in the ripe olives. Another polyphenol named ligstroside (deacetoxy-ligstroside aglycon), which contributes to the pungent odor of extra virgin olive oil, was identified in the olive fruit [35] (Figure 1). Different parts of olive fruit including seed, peel, and especially the pulp also contain oleuropein, demethyloleuropein and verbascoside, but nuzhenide is only detected in the seed [26] (Figure 1). The bitterness of the olive fruit is mainly attributed to the occurrence of oleuropein and it has to be removed in table olive processing [30]. The alkali (NaOH) treatment hydrolyses oleuropein into β-(3,4-dihydroxyphenyl) ethanol), oleoside 11-methylester and oleside making the olive palatable [21]. The detailed description of the main classes of olive phenolics is given in Table 5.

Oxidative stress is considered as one of the major factors causing different diseases such as cancer, aging, inflammation and atherosclerosis [40]. It is now widely accepted that the risk of oxidative damage can be reduced by the high intake of plant-based antioxidants. In this regard, olive polyphenols, being free radical scavengers, contribute positively towards skin health by preventing the oxidative damage linked with the formation of wrinkles and other such disorders such as skin dryness and hyperproliferation. In a clinical study, it has been shown that olive intake increases polyphenols and total antioxidant potential (TAP) in plasma, thus indicating that olive polyphenols have good bioavailability, which is in accordance with their antioxidant efficacy [24]. Olive extracts contain 73.25% maslinic and 25.75% oleanolic acids, which potentially have cancer chemopreventive activity [41]. The unripe olive fruit extract has been shown to possess the calcium channel blocking activity, considered to be responsible for its effectiveness in cardiovascular disorders like hypertension [42]. Similarly, olive fruit extract has been shown to contain a combination of laxative and antidiarroeal activities mediated through the presence of cholinergic and calcium channel blocking constituents, respectively [43,44].

### 3.2. Olive Oil

Olive oil is widely used for food preparations (as salad oil, cooking oil, in frying and pasta sauces), in cosmetics and the pharmaceutical industry [45]. In the olive fruits, oil is mainly concentrated in the pericarp (96–98%). The formation and accumulation of oil in the drupe, a rich reservoir of many classes of lipids, is possibly the reason why the oil has a unique flavour and fragrance. The olive flesh components are transformed to the oil, which mainly consists of two components, namely saponifiables and unsaponifiables. The former, comprising triacylglycerols (TAG), partial glycerides, esters of fatty acids or free fatty acids and phosphatides, represent nearly 98% of the oil chemical composition, while the later, consisting of mainly minor components such as tocopherols, phytosterols, coloring pigments and phenolics, contribute around 1–2% of the oil composition [25]. The oil triglycerides are mainly represented by monounsaturates (oleic acid), alongwith small amount of saturates and considerable quantity of polyunsaturates (mainly of linoleic acid) [46].

Several public-health based studies have revealed that the traditional “Mediterranean diet”, which includes VOO as one of the most important food ingredients, is strongly linked with the reduced prevelance of cardiovascular diseases and certain cancers [47,48]. The nutritional value and health functions of VOO are ascribed to the presence of large amount of monounsaturated fatty acids (MUFAs) such as oleic acid and valuable minor components including aliphatic and triterpenic alcohols, sterols (mainly β-sitosterol), hydrocarbons (squalene), volatile compounds, tocopherols (chiefly α-tocopherol), pigments such as chlorophylls, carotenoids (β-carotene and lutein) and antioxidants [49].

In 2004, the Food and Drug Administration (FDA) of the USA allowed a claim on olive oil labels concerning “the benefits on the risk of coronary heart disease of eating about two tablespoons (23 g) of olive oil daily, due to the MUFAs in olive oil” [50]. Oleic acid (C18:1), the principal fatty acid in oilive oil, is claimed to increase the plasma high density lipoprotein (HDL) cholesterol and apoprotein A1 and decrease the low density lipoprotein (LDL) cholesterol and apoprotein B [51]. For this reason, this fatty acid (*i.e.*, olieic acid) can prevent cardiovascular diseases that are the major cause of mortality in industrialized countries [52]. If these beneficial effects of olive oil can be attributed only to MUFAs contents, any type of high oleic acid oils such as rapeseed oil, or any MUFAs-rich food would have shown the same health benefits; this indicates that olive oil’s effects are due to more than MUFAs contents. It has been suggested that the phenolic profile of olive oil is likely to have far greater benefits on blood lipids and oxidative damage than those shown by MUFAs [10]. Based on this evidence, olive oil can be categorized as a functional food that besides having a high level of oleic acid, contains other medicinally important minor components with multiple biological activities [53].

#### 3.2.1. Fatty Acid Composition of Olive Oil

Fatty acids present in olive oil include palmitic (C_16_:0), palmitoleic (C_16_:1), stearic (C_18_:0), oleic (C_18_:1), linoleic (C_18_:2), and linolenic (C_18_:3). Myristic (C_14_:0), margaric (C_17_:0) and gadoleic (C_20_:1) acids are found in trace amount (Table 6). Also traces of 11-*cis*-vaccenic and eicosenoic acids have been detected using C-13 Nuclear Magnetic Resonance (^13^C-NMR) spectroscopic approach [18].

Almost all the cultivars of olive oil have C_16_:0, C_18_:0, C_18_:1 and C_18_:2 as the main components; C_16_:1, C_18_:3, and C_20_:0 are present in small amounts, while C_22_:0, C_20_:1, and C_24_:0 are at levels often less than 0.2%. The principal component is always the oleic acid, contributing about 55–75% of the total fatty acids. Some parameters such as the area of production, the latitude, the climate, the variety, and the stage of maturity of the fruit greatly affect the fatty acid composition of olive oil. For example, varieties of olive oil from Greece, Italy, and Spain are low in linoleic and palmitic acids but they have a high percentage of oleic acid, while the Tunisian olive oil is high in linoleic and palmitic acids and low in oleic acid [57].

Polyunsaturated fatty acids (PUFAs) with 18 carbon (C18) atoms such as linoleic (18:2 ω-6), and α-linolenic (18:3 ω-3) are known as essential fatty acids (EFAs) in human nutrition. These fatty acids, although regarded as an indispensable component for cell structure and development and function, cannot be synthesized by the human body. The intake of PUFA is necessary through diet, and should account for only 6–8% of calories from fat [25]. At the same time, the consumption of saturated fatty acids should be moderate (approximately the same amount as polyunsaturates, with a ratio of 1:1). Saturated fatty acids increase plasma cholesterol level and acts as “promoters” of certain cancer development (e.g., colon, breast, and perhaps uterus and prostate). Nutritionists recommend a balanced lipid intake corresponding to a total amount of fats equal to 25 to 30% of total calories with a ratio in fatty acids as follows: 1-Saturates (6–8%), 2-Monounsaturates (12–14%), 3- Polyunsaturates as a ω-6 (6–7%), and 4-Polyunsaturates as a ω-3 (0.5–1.5%) [25].

The two series “ω-6 and ω-3″ are, however, in contrast with each other in many aspects. Therefore, it seems important that they be present in a correct ratio in the diet, because an excess of linoleic acid can prevent the endogenous synthesis of the long chains of α-linolenic acid (eicosapentaenoic acid and docosahexaenoic acid) with consequent damage to the body. The World Health Organization recommends a ratio of 5:1 to 10:1 for ω-6 to ω-3. The ratio between the ω-6 and the ω-3 series is very important, especially during growth, because the long-chain ω-3 series are fundamental for brain and retina development. Other important functions associated include anti-cancer, antiplatelet aggregation, anti-inflammatory, and protection against dryness of the skin. The given recommended ratio is found in olive oil, whereas the same cannot be established for other vegetable oils, with the exception of linseed and soy oils [25].

It is widely accepted that the oils with higher levels of MUFAs and lower in saturated fatty acids (SFAs) are superior due to the proven beneficial effect of MUFAs on serum cholesterol levels. Table 7 shows fatty acid groups of olive oil in comparison with other edible oils. The relatively high content of MUFAs and less SFAs and considerable EFAs impart olive oil a high nutritional status, while extra virgin olive oil, extracted directly from olive fruit through mechanical means, has appreciable antioxidant activity and medicinal benefits due to the presence of an array of high-value minor components such as phenolics [9,10,37,48].

#### 3.2.2. Olive Oil Phenolics

Plant phenolic compounds are well known secondary metabolites. These can be synthesized naturally by plants in response to stress conditions such as infection, wounding, and UV radiation [58]. There are at least 30 phenolic compounds detected in olive oil belonging to the hydrophilic group [48]. The phenolic composition of olive and olive oil is very complex and the average concentration of these compounds depends on several factors including maturation stage, part of the fruit, variety, season, packaging, storage, climatologic conditions and the degree of technology used in its production [59]. If measured colorimetrically as total phenols in the methanolic extract of oil, their content may range between 40 and 900 mg/kg [60]. Biophenol compounds have potential antioxidants power and play an important role in the chemical, organoleptic and nutritional properties of the virgin olive oil (VOO) and the table olives. The main classes of phenols in virgin olive oil are phenolic acids, phenolic alcohols, hydroxy-isocromans, flavonoids, secoiridoids and lignans. The phenolic acids were the first group of phenolic compounds found in VOO; these compounds together with phenyl-alcohols, hydroxy-isochromans and flavonoids [61], are present in small amounts in VOO [62], while secoiridoids and lignans are the most prevalent phenolic compounds of oil.

Several phenolic acids, such as gallic acid, protocatechuic, *p*-hydroxybenzoic, vanillic acid, caffeic acid, syringic, *p*- and *o*-coumaric acid, ferulic acid, and cinnamic acid have also been determined and quantified in VOO (Figure 1) [37]. The contents of phenolic acids is often lower than 1 mg per kg of olive oil [63]. Secoiridoids are derivatives of oleuropein, demethyloleuropein and ligstroside. The most abundant secoiridoids of VOO are the dialdehydic form of decarboxymethyl elenolic acid linked to hydroxytyrosol (3,4-dihydroxyphenyl-ethanol) or *p*-hydroxyphenyl-ethanol (3,4-DHPEA or *p*-HPEA) (3,4-DHPEA-EDA or *p*-HPEA-EDA) and an isomer of the oleuropein aglycon 3,4-dihydroxyphenylethanol linked to elenolic acid (3,4-DHPEA-EA). Oleuropein and ligstroside aglycon and their dialdehydic forms were also detected as minor hydrophilic phenols of VOO [64,65]. The 3,4-dihydroxyphenyl-ethanol (3,4-DHPEA) and *p*-hydroxyphenyl-ethanol (*p*-HPEA) are the main phenolic alcohols of VOO; their concentration is usually low in fresh oils but increases during oil storage [66] due to the hydrolysis of VOO secoiridoids such as 3,4-DHPEA-EDA 3,4-dihydroxyphenyl-ethanol linked to dialdehydic form of elenolic acid, *p*-HPEA-EDA (*p*-hydroxyphenylethanol linked to dialdehydic form of elenolic acid) and 3,4-DHPEA-EA (3,4-dihydroxyphenyl-ethanol linked to elenolic acid) into hydroxytyrosol (3,4-dihydroxyphenylethanol) (3,4-DHPEA) and tyrosol (*p*-Hydroxyphenylethanol) (*p*-HPEA) [67]. Flavonoids such as luteolin and apigenin were also reported as phenolic components of VOO [68].

Lignans are another group of phenols found in VOO [64]; in fact, they have been recently isolated and (+)-1-acetoxypinoresinol and (+)-1-pinoresinol were characterized as the most concentrated lignans in VOO. Brenes *et al.* [69] have confirmed the occurrence of these compounds in Spanish VOO. The same author reported that the lignans concentrations discriminated the oils produced from Picual to the others virgin olive oils extracted from Hojiblanca, Coricabra and Arbequina varieties [69]. Hydroxy-isochromans is a new class of phenolic compounds of extra-virgin olive oil and the presence of 1-phenyl-6,7-dihydroxy-isochroman and 1-(39-methoxy-49-hydroxy) phenyl-6, 7-dihydroxy-isochroman has been shown in several samples [70].

Overall, a large number of phenolic compounds have been isolated from VOO, which can be further classified into **Phenolic acids and derivatives**, such as vanillic acid, syringic acid, *p*-coumaric acid**,** *O*-coumaric acid**,** gallic acid, caffeic acid, protocatechuic acid, *p*-hydroxybenzoic acid, ferulic acid, cinnamic acid, 4-(acetoxyethyl)-1,2-dihydroxybenzene, benzoic acid, hydroxy-isocromans [12,37,62,70], **Secoiridoids**, such as dialdehydic form of decarboxymethyl elenolic acid linked to 3,4-Dihydroxyphenyl-ethanol (3,4-DHPEA) (3,4-DHPEA-EDA) dialdehydic form of decarboxymethyl etenolic linked to *p*-hydroxyphenyl-ethanol *p*-HPEA (*p*-HPEA-EDA) oleuropein aglycon 3,4-dihydroxyphenyl-ethanol linked to elenolic acid (3,4-DHPEA-EA) ligstroside aglycon, oleuropein, *p*-HPEA-derivative, dialdehydic form of oleuropein aglycon, dialdehydic form of ligstroside aglycon [64,71,72], **Lignans**, (+)-1-acetoxypinoresinol, (+)-pinoresinol [64], **Flavones**, such as apigenin, luteolin [68] and **Phenolic alcohols** such as, 3,4-dihydroxyphenyl-ethanol (3,4-DHPEA), *p*-hydroxyphenyl-ethanol (*p*-HPEA) and 3,4-dihdroxyphenyl-ethanol-glucoside [73].

##### 3.2.2.1. Biological Activities and Potential Health Benefits of Olive Oil Biophenols

Reactive oxygen species (ROS), formed as a result of oxidative stress, are known to be responsible for the development of some diseases targeting lipids, proteins and deoxyribonucleic acid (DNA) in living organisms. Diseases attributed to ROS include, for example, aging, arteriosclerosis, cancer and neurodegenerative diseases such as Parkinson’s [74].

Mostly the therapeutic potential of VOO is attributed to its antioxidant compounds. In animal systems, olive oil phenolics showed their antioxidant activities *in vivo* [75], and delayed the progression of atherosclerosis [76]. In fact, olive oil phenolic compounds have good bioavailability in humans, even from small doses (22 g per day) [77], which is lower than those reported in the Mediterranean diet (30–50 g per day). The two main phenolic compounds in olive oil, tyrosol and hydroxytyrosol, are absorbed dose-dependently from olive oil [75,77]; for this reason, they can function as useful indicator of olive oil consumption, and monitoring of the compliance in clinical studies [53]. Tyrosol and hydroxytyrosol are present in plasma and urine in their glucuronide conjugated forms (around 98%), suggesting an extensive first pass intestinal/hepatic metabolism of the ingested primary forms [78,79].

Several investigations both in men and women pointed out that replacing SFAs by MUFAs in the diet can lead to a decline in blood pressure [80,81]. Olive oil was more effective in decreasing blood pressure in hypertensive patientsthan PUFAs-high diets [80]. The effect of two similar MUFAs-rich diets (olive oil and high-oleic sunflower oil) in hypertensive women patients have been studied by Ruız-Gutierrez *et al.* [82], who observed that the diet rich in olive oil induced a significant reduction of blood pressure, which was also confirmed by Fito *et al.* [83]. The beneficial effects of olives and its oil and phenolic compounds on blood pressure could be considered through their protective effect on the vascular endothelial function [53] and presence of calcium antagonist constituents. Furthermore, an olive oil rich diet can reduce the risk of breast cancer [84–86]. Likewise, antitumor effects of olive have been reported for different organs of the body such as the pancreas [87], oral cavity [88], oesophagus [89], colon-rectum [90], prostate, [91], and lung [92]. In animal studies, the protective effect of olive oil against the UV rays on the skin has also been shown [93].

In addition to the antioxidant potential, the biological activities of olive oil phenolics on enzymes have been tested in a variety of cellular models (e.g., platelets, leukocytes, and macrophages) relevant to human pathology. Most olive oil phenolics are amphiphilic and possess the ability to modulate enzymes such as cyclo- and lipoxygenases, NADPH oxidase, and nitric oxide synthase, which are involved in key functions of those cells. Hydroxytyrosol was found to considerably inhibit chemically-induced *in vitro* platelet aggregation, the accumulation of the pro-aggregant agent thromboxane in human serum, the production of the pro-inflammatory molecules and leukotrienes by activated human leukocytes, and to inhibit arachidonate lipoxygenase activity [94–97].

The protective effects of olive against the chronic and degenerative diseases are attributed to the biophenol components, particularly, hydroxytyrosol rather than to the unsaturated fatty acids content of the olive oil. These protective effects of oil can be attributed to reduction of oxidized LDL [98–100]. Other potential mechanisms include inhibition of platelet aggregation by hydroxytyrosol [95], the anti-atherogenic activity [101], inhibition ofthe changes of DNA bases caused by peroxinitrites [102] and the reduction of free radical production in the faecal matrix [64], and increase in the ratio between the reduced and oxidized forms of glutathione [103]. Moreover, a protective effect against the inflammation has been shown in the animal model [104,105]. Also, several of the beneficial aspects of the olive against cardiovascular diseases exist via its vasodilatory, anti-platelet aggregation, anti-inflammatory, antioxidant and antimicrobial properties and are associated with its oleuropein component [4,48]. Oleuropein, vanillic and *p*-coumaric acids can also inhibit the growth of some bacteria, such as *Escherichia coli*, *Klebsiella peneumoniae* and *Bacilluscereus in vitro* [106], and in the presence of 6 mg/mL oleuropein, production of aflatoxin can be decreased greatly [107]. *In vivo* studies to confirm whether oleuropein possesses antimicrobial activities in the human body are still under study [4]. The multiple biological activities and potential health benefits of olive oil/olives biophenolsare are presented in Table 8.

#### 3.2.3. Volatile and Aromatic Compounds

Olive oil, compared to other vegetable oils, is distinguished by a characteristic aroma and flavour. These sensory characteristics, together with high nutritional value are the main features that have resulted in the increase of virgin olive oil consumption in recent years [11]. Analysis of volatile components can be used as an indicator to check the quality of olive oil [140], in terms ofdetection of adulterants [141], and magnitude ofpossible off-flavours produced [142], or to determine the variety of olive. Cultivar, geographic region, fruit maturity, processing methods and parameters influence the volatile composition of olive oil [143].

Approximately 280 compounds have been identified in the volatile fraction of virgin olive oils [57]. The distinctive aroma of virgin olive oil is attributed to a wide array of compounds of different chemical classes, such as aldehydes, alcohols, esters, hydrocarbons, ketones, furans and, probably, others unidentified yet (Table 9) [144–146]. Several chemical factors such as volatility, hydrophobic character, type and the position of functional groups seem to be more related to the odour intensity of a volatile compound than its concentration. Volatile compounds found in virgin olive oil are mainly produced in plant organs by the oxidation of fatty acids through intracellular biogenic pathways [145,147]. Some of these volatile compounds are present in the intact tissue of the fruit and others are formed during disruption of cell structure during virgin olive oil production due to the enzymatic reactions in the presence of oxygen. It is generally agreed that endogenous plant enzymes, through the lipoxygenase pathway, are responsible for the positive aroma perceptions in olive oil, whereas chemical oxidation and exogenous enzymes, usually from the microbial activity, are associated with sensory defects [145,147,148]. Moreover, the presence of minor volatile compounds may provide useful quality markers and lead to better understanding of the formation or degradation of the major volatile compounds [8,145].

#### 3.2.4. Phytostrols

Plant sterols, also called phytosterols, include a major proportion of the unsaponifiables in vegetable oils. They are biosynthetically derived from squalene and form a group of triterpenes [150]. Total phytosterols content in olive oil varies between 1000 and 2300 ppm [16,151], and the amount of desmethylsterol components such as cholesterol, brassicasterol, stigmasterol, campesterol, delta-7-stigmastenol and apparent β-sitosterol in olive oil (% total sterols) are < 0.5 < 0.1 < campesterol < 4.0 < 0.5 and ≥ 93.0%, respectively [11].

**Figure 2:**
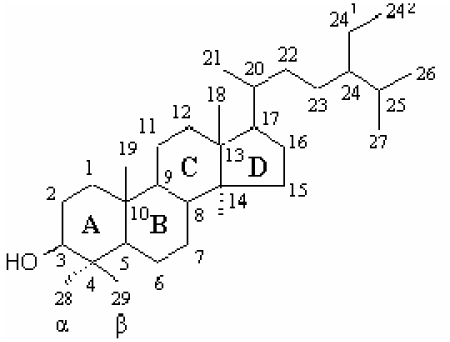
Basic structure of a sterol with standard carbon numbering according to the IUPAC [155].

The amount of sterols in the refined olive oil is considerably reduced as the refining process causes significant losses of sterols, which may be as high as 25% [152]. Phytosterols are structurally similar to cholesterol but with some modifications (Figure 2). These modifications involve the side chain and include the addition of a double bond and/or methyl or ethyl group [153]. In crude olive oil, the most common phytosterols are sitosterol (*ca*. 90%) and stigmasterol [154]. Sterol composition and content of olive oil are affected by cultivar, crop year, degree of fruit ripeness, storage time of fruits before oil extraction and the method of oil extraction [57].

Phytosterols can be classified into three classes, namely 4-desmethylsterols (without methyl group), 4-monomethylsterols (one methyl group), 4, 4′-dimethylsterols (triterpene alcohols; two methyl groups) [156] based on the presence or absence of methyl groups at the C4 position in the A rings (Figure 2). 4-desmethylsterols include all of the common phytosterols with a 28- or 29-carbon skeleton, but also cholesterol with a 27-carbon skeleton [157] (Figure 2). It is classified into Δ^5^-sterols, Δ^7^-sterols and Δ^5,7^-sterols [157,158].

Methylsterols (4-monomethyl- and 4, 4′-dimethylsterols) are synthesised at an early stage in the biosynthetic pathway and are precursors of 4-desmethylsterols [159]. 4-monomethylsterol includes Citrostadienol, Obtusifoliol, Cycloeucalenol and Gramisterol. 4, 4′-dimethylsterol, can be categorised into 24-Methylenecycloartanol, Cycloartenol α-Amyrin and β-Amyrin. The effective dosage for phytosterols to decrease LDL-cholesterolin blood is as high as 8% to 15% is 1.5 to 3 g per day. The main mechanism of this action is the interference with the solubilisation of the cholesterol in the intestinal micelles so decreasing the absorption [160]. Also, they have some anti-cancer effects in colon, breast and prostate [161], possess anti-inflammatory properties [160] and act as immune system modulators [162]. There is no evidence of any side-effect and mutagenic activity of phytosterols in the *in vitro* studies [163] or subchronic toxicity studies in animals [164]. The only limitation is that they can interfere with the absorption of carotenoids, but this can be compensated in the diet by adding these compounds in appropriate amounts [160].

The amount and phytosterol classes of seed, pulp and whole olive fruit oil is different. Seed oil has a higher concentration of total 4-desmethylsterols (2.3-fold higher), sitosterol, campesterol, chlerosterol, Δ^5–24^-stigmastadienol, Δ^7^-stigmastenol and Δ^7^-avenasterol compared to other parts oils. Usually, pulp and whole olive fruit oil have the same amounts of 4-desmethylsterols. In this regard, the amount of 4, 4′-dimethylsterols and cycloartenol, 24-methylenecycloartanol in seed oil is low but β-amyrin, butyrospermol content compared with other extracted oils is high. In general, pulp and whole olive fruit oil have almost the same concentration of 4, 4′-dimethylsterols [165].

#### 3.2.5. Tocopherols

Tocopherols are considered as the most important lipid soluable natural antioxidants, which prevent lipid peroxidation by scavenging radicals in membranes and lipoprotein particles [166]. Four different types of tocopherol, namely α-, β-, γ- and δ-tocopherol have been reported in olive oil. The amount of main component, α-tocopherol, varies from a few ppm up to 300 ppm [167]. The significant concentration of α-tocopherol in VOO supports itsideal E/PUFAs ratio. The ratio E/PUFAs can be described as the milligrams of vitamin E per gram of polyunstaurated fatty acids. This ratio which should never be less than 0.5, is rarely found in seed oils, however in VOO it is in the range of 1.5 to 2.0 [25]. The concentration of β-, γ- and δ-tocopherols are presented from traces to 25 ppm [168–169]. However, the tocopherols contents seem to be reduced during ripening fruits, refining and hydrogenation process [57].

These compounds are known to contribute to the antioxidant capacity of olive oil [108], as well as enhance oil stability during frying by protecting it from thermo-oxidative degradation [49]. Also tocopherols in virgin olive oils act not only as lipid radical scavengers, but also prevent the photoxidation by reacting with singlet oxygen by physical quenching or by chemical reactions [170]. Therefore, they increase oxidation stability of oils during storage due to preventingfrom the light [171]. Moreover, α-tocopherol defends the body against free radical attacks [172–174], and prevents skin disorders, cancer and arteriosclerosis [175–177]. However, the nature of this contribution isnot yet fully understood. Some researchers have demonstrated a synergistic relationship between the antioxidant actions of some phenolics and tocopherols [178].

#### 3.2.6. Colouring Pigments

Olive oil, like other vegetable oils, contains considerable amount of pigments such as chlorophylls and carotenoids. Chlorophylls are encountered as pheophytin. Pheophytin α concentration in olive oil ranges from 3.3 to 40 ppm, while pheophytin *b* and chlorophyll *b* are present in trace amounts andchlorophyll *a* has not been detected [179]. The main carotenoids present in olive oil are β-carotene (0.3–4.4 ppm) and lutein (trace-1.4 ppm) [180]; for instance, the concentration of carotenoids in Spanish olive oils is 3.1–9.2 mg/kg [181].

Chlorophyll, a photosensitizer, may initiate oil oxidation in olive oil when exposed to light by converting ground state triplet oxygen (^3^O_2_) to highly reactive excited state singlet oxygen (^1^O_2_*) [182]. It is interesting to note that the photoxidation reaction (light-induced oxidation) proceeds about 1000 to 1500 times faster than the common triplet oxygen oxidation [183]. β-carotene quenches singlet oxygen [183], so enhances oxidative stability against light-induced oxidation (photo oxidation). It has also been suggested that the pigment absorbs light which would otherwise excite sensitizers, therefore reducing initiation reactions [184]. The effect of β-carotene during oxidation in the dark, where reactions are not initiated by pigment sensitization, depends on the conditions under which the reactions occur. Whether β-carotene is an antioxidant or a prooxidant and how effective it is, depends on its own concentration along with oxygen [185], as well as the chemical environment where the reaction occurs [186]. Lutein has an antioxidant effect and works in combination with lycopene, as a highly active agent against skin aging and cancer risk. An adequate intake of carotenoids derived from vegetable sources including that of VOO can act as a decisive skin protector factor [25].

#### 3.2.7. Squalene

Squalene is a polyunsaturated triterpene comprising of six isoprene units and acts as a biochemical precursor of cholesterol and other steroids. It is widely produced by both plants and animals and iswidespread in nature, especially among olives, shark liver oil, wheat germ, and rice bran [187]. Thus, in addition to being synthesized within cells, it is consumed as an integral part of the human diet. Squalene content in olive oil is especially high, up to 0.7% (7 mg/g), compared to other oils and human dietary fats [188]. Only rice bran oil contained significant quantities (332 mg/100 g) [189] when compared with a large number of other seasoning oils.

Also, squalene is a major component (around 40%) of the oil unsaponifiable fraction, the material left after saponification with an alkaline hydroxide and extracted with a solvent (e.g., diethyl ether) [1]. All plants and animals including humans are capable of producing squalene. In humans, squalene is synthesized in the liver and the skin, transported in the blood by very low density lipoproteins (VLDL) and LDL, and secreted in large quantities by the sebaceous glands [190,191].

Other hydrocarbons have also been found in VOO, such as 6, 10-dimethyl-1-undecene, various sesquitterpenes, the series of *n*-alkanes from C14 to C35, *n*-heptadecene and *n*-9-alkenes [183]. Squalene has been considered to be an important component in the diet of Mediterranean people due to its chemopreventative potential against cancer. Levels of squalene (a sterol precursor) in the body achieved by including olive oil in the diet (around 40 g per day, a common value for people in Mediterranean countries) may have a considerable inhibitory effect on cancer development [192].

Special attention has been directed to squalene, present in a notable concentration in virgin olive oil nonsaponifiable fraction, which makes it similar to the composition of sebum. Squalene is found in high amounts in sebum (around 12% of its composition) and acts as a potent scavenger of singlet oxygen, inhibiting the lipoperoxidation induced by ultraviolet (UV) radiations [193], thus having anti-neoplastic influence on the colon, breast, and prostate, it seems to have immune-stimulating properties and it can inhibit the development of various tumors [194]. Also it has major protective effect against skin cancer, probably by scavenging singlet oxygen generated by UV light [189]. The oral intake as well as the external use of olive oil has been shown to provide photoprotection to the skin [195]. Such photoprotective effects of VOO against skin might be mainly correlated to the presence of notable amounts of squalane, which exerts antioxidant properties at the cutaneous level against solar rays thus behaving as a biological filter of singlet oxygen [25,64]. The presence of considerable amounts of squalane along with α-tocopherol and carotenoids in VOO provides an interesting aspect and supports the topical use of this valuable oil as an ingredient in cosmetics and dermo-potective creasems [25].

Moreover, squalene may act as a sink for highly lipophilic xenobiotics, assisting in their elimination from the body [196], and is frequently used in the preparation of stable emulsions as either the main ingredient or secondary oil [197,198]. Squalene emulsions have been used for various applications, especially for the delivery of vaccines, drugs, and other medicinal substances [199]. Since squalene is well absorbed orally, it has been used to improve the oral delivery of therapeutic molecules. Today, there are claims that squalene can enhance the quality of life, if taken continually and orally [194].

#### 3.2.8. Triterpene Dialcohols

There is another group of compounds present in the unsaponifiables fraction of olive oil called triterpene dialcohols, which are co-chromatographed with 4-desmethylsterols [57] and present in the range of 500–3000 mg/kg [200]. Erythrodiol and uvaol are the two main triterpene dialcohols present in olive oils [16]. Their concentration is mainly affected by cultivar. The amount of total erythrodiol ranges from 19–69 mg/kg [201], and concentration of free erythrodiol is usually lower than 50 mg/kg. One way to distinguish between virgin olive oil and solvent extracted olive oil is on the basis of the amount of erythrodiol and uvaol [202]. Aaccording to IOOC standard [16], the total amount of these two compounds is ≤4.5% of total sterols in VOO, while in olive–pomace oil (solvent extracted oil) this limit is ≥4.5%.

### 3.3. Olive Leaf

Historically, olive leaves have been widely used as a remedy for the treatment of fever and other diseases like malaria [203–205] in European and Mediterranean countries such as Greece, Spain, Italy, France, Turkey, Palestine, Morocco and Tunisia. As a dietary component, the leaves have been consumed in the form of an extract, a whole herbor powder [206]. Olive leaves contain many potentially bioactive compounds that may have antioxidant, anti-hypertensive, anti-inflammatory, hypoglycaemic and hypocholesterolemic properties [206].

Several reports demonstrated that olive leaves can decrease blood pressure, increase blood flow in the coronary arteries [138,207,208], decrease arrhythmia and prevent intestinal muscle spasms [209]. The leaves also possess antimicrobial properties against some microorganisms such as bacteria, fungi, and mycoplasma [119,124,136,210–213].

These potential health benefits of olive leaves are mostly related to low molecular weight polyphenols such as oleuropein (up to 60–90 mg/g dry leaves weight), hydroxytyrosol, tyrosol, tocopherol, elenolic acid derivatives, caffeic acid, *p-*coumaric acid and vanillic acid as well as flavonoids: luteolin, diosmetin, rutin, luteolin-7-glucoside, apigenin-7-glucoside, and diosmetin-7-glucoside [214–216]. Moreover, the combined phenolic compounds have significantly higher antimicrobial activity than those of the individual phenolics [124].

Due to these activities and valuable biophenol compounds, usage of whole olive leaf and olive leaf extract has increased rapidly in both the pharmaceutical and food industries as food additives and functional food materials [204,217]. The whole leaf extract is recommended to achieve health benefits due to the presence of additive and/or synergistic effects of their phytochemicals [209].

### 3.4. Olive by-Products

As a result of olive processing, a huge quantity of olive by-products are produced. Typically, an olive oil processing industry produces approximately 35 kg olive cake and 440 L olive mill waste water (OMW) per 100 kg of processed olives [105]. A major environmental problem in the Mediterranean countries is the disposal and/or treatment of the large quantities of OMW and olive cake produced during olive oil processing [218]. The high-polluting power of OMW is generally associated with the high biochemical oxygen demand, chemical oxygen demand, total solids, organic carbon and the slight acidic character [219]. With the current trends in functional foods, appropriate techniques should be sought for the isolation of valuable bioactives from the olive residues for potential uses.

#### 3.4.1. Olive Cake

Although olive cake is an economical biomass present in large quantities, it causes some environmental problems for Mediterranean countries [218]. For this reason, olive cake is often consumed as fuel, fertilizer and animal feed [220]. Olive cake is considered as a rich source of phenolic compounds with a wide array of biological activities. In fact, three aspects of antioxidant attributes have been investigated in olive cakes; antioxidant capacity [221,222], anti-radical activities and radical scavenging activities [58,218,223]. Accoording to some studies, hydroxytyrosol [224], oleuropein [225], tyrosol [226], caffeic acid [218], *p*-coumaric acid, vanillic acid [227], verbascoside, elenolic acid [225], catechol [228] and rutin [29] are the main phenolic compounds in olive cake. However, little work has been doneuntil now on therecovery of phenolic compounds from olive cake as a potential source of bioactives for potential uses in the pharmaceutical and nutraceutical industries [229]. Recently, Aludatt*et al.* [230] optimized some parameters for extraction of phenolic compounds from olive cake and reported that the highest total phenolic compounds and antioxidant activity were achievedusing methanol at 70 °C for 12 h. Also the major free phenolic compounds in full-fat and defatted olive cake were protocatechuic acid, sinapic acid, syringic acid, caffeic acid, and rutin. Table 10 shows the bound phenolic compounds profiles of extracts from full-fat and de-fatted olive cake derived in sequential extractions [230].

There is small variation observed for the amounts of the phenolic compounds (extracted by using alkaline hydrolysis) between full-fat and defatted olive cake except for the hesperidin and quercetin, which are only detected in defatted olive cake. Efforts should be made to explore the potential uses of this olive biomass in pharmaceutical, nutraceutical, functional food products not only for value addition but also to decrease the effect of olive oil production on the environment.

#### 3.4.2. Olive Oil Mill Waste Water

Olive oil mill waste water (OMW) constitutes exert a serious problem with severe negative impact on soil and water quality, and thus on agriculture, environment and health [231]. On the other hand, the amount ofbiophenol compounds in olive oil is 2% of the total phenolic content of the olive fruits, the remaining 98% being lost in olive mill waste [229]. OMW, as a rich source ofbiophenol compounds with multiple biological activities, and free radical-scavenging and metal-chelating properties, have been shown to be more effective antioxidants *in vitro* than vitamins E and C on a molar basis [232]. GC–MS analysis revealed that the free phenols could be recovered from OMW by a simple liquid–liquid extraction process. The phenolic extract is composed of hydroxytyrosol as the major compound (66.5%), while tyrosol, cafeic acid, *p*-coumaric acid, homovanillic acid, protocatechuic acid, 3, 4-hihydroxymandelic acid; vanillic acid and ferulic acid are among others, which means that OMW extract can be a natural source of useful substances [233]. Some of the important bioactivities associated with OMW polyphenols, include antioxidant effect on intestinal human epithelial cells [234], anti-inflammatory activity through inhibition of 5-lipoxygenase [96], antiviral [28], molluscicidal [235], antibacterial and antifungal [106], cardioprotective, anti-atherogenic [236] and anti-tumour [64] activities.

The anti-microbial activity of OMW has been studied by Gonzalez *et al.* [237] and was reviewed by Moreno *et al.* [238], who attributed antimicrobial and phytotoxic activities to minor biophenols and phenolic acids [28]. A non-polar extract of OMW did not show any activity on the bacteria, so its bioactivity was correlated with the hydrophilic components such as phenolic content of OMW [239], and its anti-bacterial activity was more marked on Gram-positive than on Gram-negetive bacteria. Perez and colleagues [239] found that Ethylacetate and *n-*propanol OMW extracts had the strongest effect on *Bacillus meganterium.* Capasso *et al.* [240] determined the antimicrobial activity of OMW and found that hydroxytyrosol had activity just against *Pseudomonas savastanoi* but 4-methylcatechol was the strongest antibacterial compound against *Pseudomonas syringae.* Other compounds identified in OMW with antimicrobial activity were oleuropein, hydroxyltyrosol, 4-hydroxybenzoic acid, vanillic acid and *p*-coumeric acid [4], and hydroxytyrosol being more effective than oleuropein [28]. It was also observed that OMW extract can be used as natural antioxidants instead of synthetic antioxidants to protect edible oils and food products from oxidation [241].

#### 3.4.3. Olive Stone

The olive stone and seed are important by-products generated in the olive oil extraction and pitted table olive industries. The whole olive stone consists of the wood shell (stone) and the seed. In the olive oil industry, only the olive stone without seed can be recovered by filtration of solid waste. From the pitted table olive industry, the whole olive stone (stone and seed) is recovered by separation of the pulp. The main lignocellulosic components in olive stone are hemicellulose, cellulose and lignin with 21.45–27.64%, 29.79–34.35%, 20.63–25.11%, respectively [242]. Protein, fat, phenols, free sugars and phenolics are also present in considerable quantities (Table 11). The main use of this biomass is in combustion to produce electric energy or heat. Other uses such as production of activated carbon, applied for removal of unwanted colours and dyes [243], odours, tastes or contaminants such as arsenic [244] or aluminium [245], furfural production [246], and plastic filling [247], have also been cited. Besides, this bio-mass has been reported to be used as metal bio-sorbent [248], animal feed [249], and in resin formation [250].

Interestingly, the whole olive stone is a rich source of bioactive compounds. These potentially valuable compounds are nuzhenide-oleoside, nuzhenide, salidroside, which are detected only in the olive seed;verbascoside only appears in significant quantities in the seed and pulp [214], but tyrosol, hydroxytyrosol, oleuropein and diadehydic form of decarboxymethyl oleuropein (3,4 DHFEA-EDA) are found in different parts of olive including the pulp, leaves, seed and stone [248].

#### 3.4.4. Olive Wood

In view of the large amounts of wood generated from olive tree pruning that is now mostly burnt, olive wood would also be a very interesting and plentiful source of antioxidants. The isolation and radical scavenging activity of the six main components from olive wood, such as, tyrosol, hydroxytyrosol, (+)-cycloolivil, ligustroside, oleuropein and 7-deoxyloganic acid have been reported [251]. Besides having antioxidative activities, it has been reported that oleuropein and (+)-cycloolivil possess anti-platelet aggregation properties, and both compounds inhibit protein tyrosine phosphorylation, which suggests that they may prevent thrombotic complications associated with platelet hyperaggregability [252]. Recently, antioxidants have been isolated and identified in the ethylacetate extract of olive wood. A new secoiridoid compound, oleuropein-3′-methylether, together with six known secoiridoids,7′S-hydroxyoleuropein, jaspolyanoside, ligustroside 3′-*O*-β-D-glucoside, jaspolyoside, isojaspolyoside A and oleuropein 3′-*O*-β-D-glucoside, were isolated and the structures of these compounds determined by spectroscopic methods [253].

## 4. Factors Affecting Chemical Composition of Olives

The chemical composition of olives may vary depending upon different factors including agronomical factors (e.g., olive cultivar) [31], the ripening stage of the fruit [30], agroclimatic conditions [6] and irrigation management [254,255]. Several studies have already been carried out to describe the differences found between the phenolic profiles of different olive cultivars [6] as well as their distribution throughout the ripening process [30,26,256].

### 4.1. Cultivars

The composition of olives varies with cultivar and environmental conditions. Olive cultivar also affects the absolute concentration of the specific hydrophilic phenols of VOO, while the phenolic profile remains almost the same, but olive variety and harvest time have a statistically significant influence on the amount of total phenols, ortho-diphenols, as well as intensity of bitterness, while stage of ripeness in olives demonstrated a more notable effect than the species itself [257]. Many studies have been published demsonstrating considerable variations in the amounts of total phenolics among different cultivars of olives (Table 12).

### 4.2. Fruits Maturity

Phenolic compounds change qualitatively and quantitatively during growing period [30,31,260]. There are two maturation stages in olive fruits corresponding to green and black fruits. In the green maturation stage, olive fruits reach their final dimensions and their colour is yellow green. After, the green coloring pigments (*i.e.*, chlorophylls) in the skin are replaced by anthocyanins resulting the transition to a “spotted,” “purple” and “black” stage. At the stage between the yellow green and purple skin, the olives have the highest amounts of phenolic compounds, especially oleuoropein.

Much research has concentrated on olives phenolics and especially on oleuropein, which is known to be the most important individual phenolic component of olive pulp, reaching concentrations of up to 14% on a dry weight basis in young Picoline olives [30]. The amount of total phenolic compounds, especially oleuropein, decreases during fruit maturity [30,261] because during this stage esterase activity degrades oleuropein [30] and it can reach zero in some cultivars when olives are absolutely dark [21]. Reduction of oleuropein concentration is accompanied by the accummulation of two compounds, namely demethyloleuropein and elenolic acid glycoside [262].

Besides the maturation state of fruits, oleuropein concentration can be affected by the dimensions of the fruits. Generally, some species with small olive fruits have a high level of oleuropein from the initial up to the end of maturation but in another species, which produce large fruits, quantities of oleuropein is low during the maturation process

Moreover, species with high levels of oleuropein have minimum verbascoside [21], but its concentration during fruit maturationincreases steadily [259]. The amount of ligstroside in young olives is higher but it decreases during maturation. Also, some changes have been observed in the level of oleoside 11-methylester, tyrosol, hydroxytyrosol and their glucosides within fruit developing [21]. Cimato *et al.* [263] showed that with fruit ripening, hydrolysis of components with ‘higher molecular weight’ occurred, resulting in the formation of tyrosol and hydroxytyrosol. Thus, the concentration of tyrosol and hydroxytyrosol was also shown to increase with the harvesting period, which has been correlated with an evident reduction in four unidentified, but [2] resumably phenolic components.

Besides the stage of maturation, some parameters such as fly (*Bactrocera olaea*) attack have an effect on the phenolic compounds in oils extracted from olives [264]. Several other studies have discussed the effect of maturation on the quantity of phenolics in various olive cultivars [6,69,262,265–274].

### 4.3. Irrigation

Ecological factors as well as agronomic practices such as irrigation have an effect on the phenolic content of VOO. Irrigation is an essential parameter, even in fields where water is unrestricted, for achieving better production, productivity and characteristics of olive oil, because good quality olive oil cannot be obtained from olive fruit harvested under high levels of water stress [275,276]. Several authors have determined variation in the chemical composition and the sensory qualities of VOO obtained from olive trees under irrigated and rain-fed conditions [201,277]. Some showed that irrigation had a clear effect on the phenolic compounds as major substances were affected [254,255,278]. Indeed, it has been observed that the total phenol content in different olive oils and its oxidative stability decreased when the trees received alarge amount of water [279].

The effects of irrigation with fresh and saline water on olive oil quality has also been studied [258], and the results showed that irrigation with saline and fresh water-type did not have a significant effect on olive oil composition, but the amount of total phenols decreased. According to published data [280], fundamental quality parameters, such as free acidity, peroxide value, and fatty acids profile did not change in olive oil during irrigation with saline water, though polyphenols and vitamin E content increased with the saline treatments. A few studies have reported that irrigation with treated waste water can affect the olive oil composition and decrease the total phenols content [281]. However, this irrigation did not exert any notable effect on the free fatty acid and specific ultraviolet absorbance K232 and K270 of olive oil [282].

### 4.4. Technological Aspects in Olive Oil Extraction

VOO is produced by mechanical and physical processes [283]. These processes involve collection, leaves removal, washing, olives crushing, malaxition the olive paste, centrifugation with or without adding water which is named “three-phase” or “two-phase” respectively, storage, filtration and bottling. The presence of hydrophilic phenols in VOO depends on different endogenous enzymes of olive fruits and extraction conditions. During the oil mechanical extraction process, crushing and malaxation play an important role [284,285]. Several modifications such as hydrolysis of glycerides by lipases, hydrolysis of glycosides and oligosaccharides by β-glucosidases, oxidation of phenolic compounds by phenoloxidases, and polymerization of free phenols can appear during these processes [2].

#### 4.4.1. Crushing

This operation is designed to tear the fruit cells to release the droplets of oil from the inner cavity (vacuole). Crushing is an important part of extracting VOO, because it affects the physical and chemical properties of oil. Before crushing the fruits, the oil is protected inside the cell, but after crushing it contacts other constituents of the cell including enzymes, that affect the quality of oil. In the earlier days, the crushing process was carried out with a system of heavy wheels made of stones. Nowadays when the continuous extraction systems came into use the metal crusher-hammer or toothed-disc are used to grind the olives. Using the hammer increases the oil extraction yield because the intercellular structure is destroyed by using the stone mill and consequently oil droplets may be retained inside the cells, while the hammer cut the cells without destroying the intercellular structure [286]. The metal crusher may increase the yield of extraction from olives, but because of high speed it may create more emulsion than a stone crusher, therefore, the produced paste must stay longer in malaxation process. The concentration of phenols present in VOO depends on the way the olive paste is prepared [287]. Using a hammer crusher instead of a stone crusher increases the amount of total phenols components and ultimately the stronger antioxidant power in the oil, in terms of the concentration of polyphenolic compounds (Table 13). This can be ascribed to the higher temperature which is caused by the speed of the hammer crusher as well as solubilisation phenomena by which more phenolic compounds pass into the oil [286,287]. Also, when olives processed by these two crushing methods were analyzed by scanning electronic microscopy, the micrographs showed evidence that olives treated by hammer crushing system were better cut than those treated by stone mill because olive cell layers were broken and damaged by the later technique [286].

#### 4.4.2. Malaxation

Malaxation process (also called beating or kneading) is essential for increasing extraction yields. It is designed to enhance the effect of crushing and to make the paste uniform. The majority of oil in olive fruit is located in the vacuoles of mesocarp cells. During the malaxation step the small droplets of the oil, by means of slow and continuous kneading of the paste produced by metallic crusher, merge into large drops that can be easily separated by the separating apparatus [147]. This process also breaks the produced emulsion and helps to increase the oil extraction yield. The bioactives composition in olive oil can be significantly improved by various factors including malaxation temperature, time [289], and the use of microorganisms [290], or enzymes [291,292].

Addition of commercial enzyme preparations such as pectolytic, hemicellulolytic, and cellulolytic during the olive oil malaxation process resulted in degrading the cell wall of the fruit and reducing the complex of hydrophilic phenols with polysaccharides, increasing the concentration of free phenols in the olive paste and their consequent release into the oil and wastewaters through processing [293].

Oleuropein and demethyloleuropein and ligstroside hydrolyze within the crushing and malaxing step due to endogenous glycosidases and thus change to dialdehydic form of decarboxymethyloleuropein aglycone and aldehydic form of oleuropein aglycone [294].

#### 4.4.3. Decantation

After the malaxation process, the oil must be separated from the paste and vegetation water by using a decantation process. Decanters are of three types, namely: (1) oldest three-phases decanter (50–100 L of added water per 100 kg of olive paste); (2) three-phases decanter that works using less water (10–30 L of added water per 100 kg of olive paste); (3) two-phases decanter that operates without adding any water. In this section, pressure and centrifugation as an extraction system play an important role in recovering the amounts of phenolic compounds. Actually, in three phase system in order to reduce the viscosity of pastes and to separate oil easily from the solid phase sufficient water needs to be added before centrifugation [73].

Disadvantages of this process include higher amounts of waste water (1.25 to 1.75 times more water than press extraction), loss of valuable components (e.g., natural antioxidants, phenolics) in the water phase, and problems of disposal of the oil mill waste water. Therefore, pressure system that does not need to add water to the olive paste offers a large amount of phenolic compounds in comparison to those obtained by the centrifugation system [73]. Two phase centrifugation systems have evolved within the last 10 years to need less water for the separation of oil. As shown in Table 14, higher contents of phenolic compounds are obtained in the oils produced by the new two phases’ decanter as compared to the traditional three-phase decanter [295,296]. Thus, the oil produced by a two-phase system has stronger antioxidant activity (2-fold greater) and higher resistance to oxidation than that obtained by a three-phase system due to the higher amount of hydroxytyrosol as an orthodiphenol compound [297–299].

#### 4.4.4. Filtration

Filtration is the final step in olive oil processing and can be carried out with various materials or filter aids in combination with filtration hardware to improve filtration performance [300]. This process is also used for removing humidity and in this case cotton or paper filters can be used. Filtration also can affect the phenolic compounds. Using a cotton filter for removing humidity, hydroxytyrosol content in extra virgin olive oil was decreased [276,300]. However, filtration with cotton or paper plus anhydrous sodium sulphate led to an apparent increase in the phenolic content. Sometimes olive oil has a lot of suspended solids and in this case the diatomaceous earth is used for filtration purposes. Nowadays, organic materials such as cellulose fibrous materials and starch are used as filter aids. The solid residue waste after filtration with diatomaceous earth and organic filter aids may offer another source of phenolic compounds. Filtration by an organic filter aid is preferred over diatomaceous earth due to the high performance in the filtration process onlaboratory scale [300]. Thus, filtration especially dehydration can assist to improve the shelf-life and nutritive quality of olive oil in some cultivars such as Arbequina and Colombaia [301].

Different classes of the hydrophilic phenolics such as phenolic acids, phenolic alcohols, secoiridoids, lignans and flavones can be retained in the filter aids. Phenolic acids including, vanillin, vanillic, ferulic and *p*-coumaric acids have been identified in different filter aids. Similarly, phenolic alcohols, tyrosol and hydroxytyrosol in addition to secoiridoids have been found in almost all filter aids. The most important secoiridoids retained in filter aids include oleuropein aglycon, 10-hydroxy-oleuropein aglycon, decarboximethylated derivates of oleuropein aglyco, oxidation products of dialdehydic form of decarboxymethyl oleuropein aglycone and ligstroside aglycon. Lignans, pinoresinol, hydroxy-pinoresinol, and acetoxypinoresinol have also been detected. All the filters retained different flavones such as apigenin and luteolin, someunknown compounds have also been investigatedin the filter aids [300].

## 5. Potential for Recovery of Valuable Olive Natural Constituents

A more recent approach for obtaining olive mill waste (OMW) has involved the use of processing technologies to fractionate potential high-value components from olive agrowastes and residues. The recovered compounds may be broadly classified into insoluble, water-soluble and lipid solubles. One of the most important environmental problems in the Mediterranean countries, such as Spain, Italy and Greece, is the treatment and disposal of OMW. The main organic contents of OMW are sugars, nitrogenous compounds, volatile acids, polyalcohols, pectins, fats and polyphenols. During the past years, olive oil processing industries have used a continuous centrifugation system with a two-phase decanter, which separates VOO by recycling the vegetation water of the processed olives. This technology considerably decreases the volume of plant effluents and the disposal problems [238].

However, little information has been reported on the recovery of phenolic compounds from olive cake and OMW as a potential source of bioactive compounds for the pharmaceutical and nutraceutical industries [229]. In this regard some trials have been carried out by using physico-chemical or biological treatments to decrease the potential pollution load and obtain antioxidant compounds [233,302–305]. However, there are many limitations for the industrial-scale usage of the methods so far proposed. The major problems for the recovery of such valuable components are correlated to the complexity of the proposed processes, requiring water pre-treatment, or the huge costs for the purchase and the maintenance of the instruments.

One of the methods applied for recovering hydrophilic phenols from fresh OVW (olive vegetation water) is three consecutive membrane-filtration steps with decreasing cut-off values (microfiltration, ultra filtration and reverse osmosis) by a previous enzymatic treatment. Its products include crude phenolic concentrate permeate without any phenolic compounds and large amount of organic fraction that could also be re-used in the VOO-extraction process. Between membrane processes, nano-filtration is considered as an effective procedure for the removal of these compounds from waste water [306].

Another way to extract phenolic compounds is super critical fluid extraction (SFE) with carbon dioxide, which produces extracts with high antioxidant quality from spices and agricultural by-products but its phenolic yield is not appropriate. Typically, ethanolic extraction provides high yield with stronger antioxidant activity than butylated hydroxytolune (BHT), ascorbyl palmitate and vitamin E. Also, analysis by HPLC shows that hydroxytyrosol is a major phenolic compound in it [307]. Consequently, the OMW can be explored as an economical and renewable source of phenolic antioxidants. Phenolic extracts from OMW can be used as a natural alternative for commercial synthetic antioxidants or for other food and medicinal uses.

## 6. Conclusions

Consumption of olives and/or olive oil is recognized as a key factor supporting the beneficial effects of the “Mediterranean diet”. Olive oil, having been used as a nutritious food, drug, and as cosmetics for centuries by the Mediterranean people, has been a subject of much scientific interest in the last few decades, confirming its multiferous biological, therapeutic and functional food applications. Currently, due to continuing scientific evidence supported with numerious epidemiological and clinical experimental studies, the recognition of olive oil as a source of food and medicine is much acknowledged. The most important activities in olive oil are antioxidant, anti-microbial, anti-inflammatory and anti-cancer as evident from a variety of studies. Olive oil is resistant to oxidation and it has a special bitter and pungent taste. Principally, these biological activities and individual taste are due to the presence of unique bio-active compounds in the olives, namely phenolics (e.g., oleuropein, hydroxytyrosol, verbascoside and derivatives), tocopherols and carotenoids, amongst others. Several factors, such as agronomical conditions, climate, and level of ripening, olive cultivar and type of production process have the main effects on the profile and activities of bio-active compounds in olives and olive oil. During olive oil processing, in addition to the olive oil itself, olive cake and oil mill waste water are produced, which are considered to be good sources of phenolic compounds with multiple epidemiological and therapeutic activities, thus highlighting the potential of such olive by- products for the isolation of high-value bioactives for pharmaceutical, nutraceutical and food industries.

## 7. Future Prospects

Nowadays, the significance of functional foods is growing rapidly in food science, where inquires on the bioactivity, bioavailability and toxicology of phytochemicals and their stability and interactions with other food ingredients need to be carefully studied under *in vitro* and *in vivo* conditions [308]. Indeed, olive oil by-products are a good source of phytochemicals and natural antioxidants, which could be exploited for their health promoting properties. However, in this regard, significant efforts should be rendered to isolating, purifying and recovering optimum amounts of valuable compounds, in high class of purity followed by studying their detailed and standardized biological and pharmaceutical attributes. Efforts should also be focused on the isolation and structural elucidation of novel olive phenolic lipids and other bioactives using state-of-the-art chromatographic and spectroscopic tools. If the recovery and development of novel products from the olive oil by-products is well achieved, it can help to solve the environmental problems in “Mediterranean countries” and also promote application of olive oil in the food, pharmaceutical and cosmetic industries.

Industrial processes, which could minimise the loss of bioactive compounds, need to be developed. The standardization of olives and olive oil dietary intakes, based on the known chemical composition, will help to provide sound clinical basis for assessment of potential anti-atherosclerotic, anti-hypertensive, anticancer, anti-platelet aggregation and immune modulatory functionalities of olive bioactives and thus development of olive-based functional foods and nutraceuticals.

## Figures and Tables

**Figure 3:**
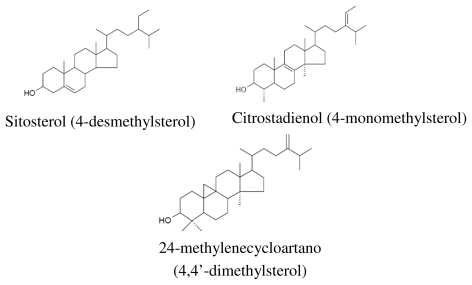
Chemical structural of some important phytosterols in olive oil [18].

**Table 1:** List of some common olive cultivars and their origins in the world [12].

Origin	Cultivar
Greece	Adramitini, Amigdalolia, Amphissis, Chalkidikis (Chondrolia), Daphnoelia Doppia, Frantoio, Gordal, Koroneiki, Karidolia, Lianolia, Patrini, Chondrolia (aka Throumbolia), Tsounati, Valanolia
Italy	Biancolilla, Bosana, Canino, Casaliva, Cellina di Nardo, Coratina Dolce Agogio, Dritta, Moraiolo, Rosciola, Pisciottana, Grignan, Ottobratica
Spain	Alfafara, Arbequina, Bical, Blanqueta, Empeltre, Farga, Gordal, Lechin, Hojiblanca, Manzanilla de Jaén, Morrut Palomar, Picual, Sevillenca Verdiell, Vilallonga
France	Aglandau, Amellau, Cayon, Germaine, Picholine, Lucques, Sabine, Salonenque Picholine, Zinzala
Portugal	Cobrancosa, Galega
Croatia	Oblica and Leccino
Tunisia	Chemlali, Chetoui, Gerboui, Meski, Oueslati

**Table 2:** Some important properties of popular cultivars, their utilization and origin [12].

Cultivar	%Oil yield	Ratio flesh to pit	Olive size (Fruits/kg)	Purpose	Shape of the fruits	Origin
Hojiblanca	23–29	4.9 and 6.6:1	230–700	Oil extraction and table olives	Regular	Spain
Verdial	22–30	6:1	220–800	Oil extraction and table olives	Ellipsoidal	Spain
Picual	23–27	3.8 and 6.1:1	270–470	Oil extraction and table olives	Prominent tip at the button end	Spain
Domat	22	-	180–190	Green table olive	Regular	Turkey
Gemlik	27	6:1	270–280	Black table olives	Pronounced tip at the end	Turkey
Memecik	22	6:1	205–215	Oil extraction	Pronounced tip at the end	Turkey
Memeli	25	7 : 1	200–210	Green-type table olives	Small tip at the end	Turkey
Conservolea	22–25	8:1	180–200	Table olive	Round to oval	Greece
Nychati Kalamon	25	8:1	220–240	Black table olive	Cylindro-conical, Curved	Greece
Chalkidiki	19	-	120–140	Green table olive, oil extraction	Pronounced tip at the end	Greece
Sevillano	14	7.3:1	70–80	Table olive	-	Spain
Ascolano	19	8.2:1	110–120	Table olive	-	Italy
Ascolana	17	-	100–180	Table olive	Spherica	Italy
Barouni	17	6.8:1	130–140	Green & black - ripe olives	-	Tunisia
Picholine marocaine	17	5:1	300–500	Table olive	-	Morocco
Arauco	22–24	7:1	125–300	Table olive and oil extraction	Pronounced tip	Argentina
Galega vulga	-	4:1	430	Black table olive	-	Portugal
Oblitza	22	6.5:1	200	Table olive	Apple and heart shape	Yugoslavia
Ladoelia	18–21	4.6:1	330	Table olive	-	Cyprus

**Table 3:** Major olive production countries in 2009 [15].

Country	Production (tonnes)	Cultivated area (hectares)	Yield (quintal/hectar)
World	18,241,809	9,922,836	18.383
Spain	6,204,700	2,500,000	24.818
Italy	3,600,500	1,159,000	31.065
Greece*	2,444,230	765,000	31.4
Turkey	1,290,654	727,513	17.740
Syria	885,942	635,691	13.936
Morocco	770,000	550,000	14.000
Tunisia	750,000	2,300,000	3.260
Egypt	500,000	110,000	45.454
Algeria	475,182	288,442	16.474
Portugal	362,600	380,700	9.524
Libya	180,000	Na	Na
Argentina	160,000	52,000	30.769

Na: not available.

* Data for Greece is for the year 2007.

**Table 4:** World wide major production and consumption of olive oil [15].

Country	Production in tons (2009)^a^	Production % (2009)	Consumption (2005)^b^	Consumption per person annually (liters/kg)^c^
World	2,907,985	100%	100%	0.43
Spain	1,199,200	41.2%	20%	13.62
Italy	587,700	20.2%	30%	12.35
Greece	332,600	11.4%	9%	23.7
Syria	168,163	5.8%	3%	7.0
Tunisia	150,000	5.2%	2%	11.1
Turkey	143,600	4.9%	2%	1.2
Morocco	95,300	3.3%	2%	1.8
Portugal	53,300	1.8%	2%	7.1
France	6,300	0.2%	4%	1.34
United States	2,700	0.1%	8%	0.56
Others	169,122	5.8%	18%	1.18

a FAOSTAT crop processed 2009 data for olive oil;

b United Nation Conference on Trade and Development;

c California and World Olive Oil Statistics.

**Table 5:** Main classes of phenolic compounds in olive fruit.

Phenolic compounds	Reference
**Flavonols** Quercetin-3-rutinoside, Luteolin-7-glucoside, Luteolin-5-glucoside, Apigenin-7-glucoside	[2,36]
**Phenolic acids** Chlorogenic acid, Caffeic acid, *p*-Hydroxybenzoic acid, Protocatechuic acid, Vanilic acid, Syringic acid, *p*-Coumaric acid, *o*-Coumaric acid, Ferulic acid, Sinapic acid, Benzoic acid, Cinnamic acid, Gallic acid	[2,12,36,37]
**Phenolic alcohols** (3,4-Dihydroxyphenyl) ethanol (3,4-DHPEA), (*p*-Hydroxyphenyl) ethanol (*p*-HPEA)	[2,31,38]
**Secoiridoids** Oleuropein, Demethyloteuropein, Ligstroside, Nuzhenide	[26,34,35,38,39]
**Hydroxycinnamic acid derivatives** Verbascoside	[26,27]

**Table 6:** Fatty acid composition (%) of olive oil from selected cultivars.

Fatty Acid	IOOC (a)	Arbequina (b)	Abosasana (b)	Koroneiki (b)	Frantoio (c)	Leccino (d)	Busa (d)
Myristic acid	< 0.05	ND	ND	ND	ND	ND	ND
Palmitic acid	7.5–20.0	17.57	17.78	11.65	10.9	13.7	12.07
Palmitoleic acid	0.3–3.5	2.41	2.12	1.07	0.89	1.32	1.02
Heptadecanoic acid	< 0.3	ND	ND	ND	0.07	ND	ND
Stearic acid	0.5–5.0	1.88	2.07	2.15	1.53	1.9	1.97
Oleic acid	55.0–83.0	58.82	64.79	75.53	78.3	75.69	74.54
Linoleic acid	3.5–21.0	12.93	12.09	8.56	6.79	5.65	8.36
Linolenic acid	< 1.0	0.63	0.54	0.26	0.49	0.161	0.66
Arachidic acid	< 0.6	0.40	0.33	0.42	0.33	0.3	0.33
Gadoleic acid (eicosenoic)	< 0.4	ND	ND	ND	0.27	ND	ND
Behenic acid	< 0.2	ND	ND	ND	0.18	ND	ND
Lignoceric acid	< 0.2	ND	ND	ND	ND	ND	ND

ND: not detected.

(a) —[16];

(b) —[54];

(c) —[55];

(d) —[56].

**Table 7:** Fatty acid groups of olive oil in comparison to some other edible oils [25].

	Saturates (%)	Monounsaturates (%)	ω-6 (%)	ω-3(%)
Butter	45–55	35–55	1.5–2.5	0.5
Lard	40–46	42–44	6–8	0.5–0.9
Olive oil	8–14	65–83	6–15	0.2–1.5
Peanut oil	17–21	40–70	13–28	**-**
Maize oil	12–28	32–35	40–62	0.1–0.5
Soyabean oil	10–18	18–30	35–52	6.5–9
Sunflower oil	5–13	21–35	56–66	**-**

**Table 8:** Biological activities and potential health benefits relating to olives/olive oil phenolics.

Biological Activity	Potential Clinical Target	References
Antioxidant activity	Cardiovascular and degenerative diseases	[101,104,108–125]
Anti-inflammatory activity	Inhibition of pro-inflammatory enzymes	[94–96,126,127]
Antimicrobial activity	Infectious diseases	[124,128–131]
Anti-atherogenic activity	Coronary heart diseases, stroke	[25,95,101,119]
Anti tumor activity	Various cancers	[84–86,95,96,132–135]
Anti platelet aggregation	Coronary heart diseases, stroke	[95,136,137]
Anti-hypertensive activity	Hypertension	[40,83,98,119,138,139]
Increased vitamin A and β-carotene activity	Antiaging/skin protection	[25]
Increased immune activity	Infectious diseases; various cancers	[25]
Anti-allergic activity		[25]
Reduction in the levels of plasma cholesterol and oxidized LDL	Coronary heart diseases	[25,98–100]

**Table 9:** Volatile compounds in olives/olive oil and their characteristics [145,149].

Attribute/Aroma	Correlated compounds
Green	methyl acetate, 1,3-hexadien-5-yne,4-methyl pentan-2-one, 2-methyl-1-propanol, (Z)-3-hexenal, hexyl acetate, 3-hexenyl acetate, (Z)-2-penten-1-ol, (E)-2-hexen-1-ol, (Z)-3-hexen-1-ol
Sweet	ethyl furan, ethyl propanoate, 1-penten-3-one, butyl acetate, hexanal, Ethyl butanoate
bitter and pungent	ethyl benzene, (E)-2-hexenal, (Z)-2-hexenal, 6-methyl-5-hepten-2-one, quinine, caffeine, alkaloids. tridecene,1-penten-3-one, 1-penten-3-one
Undesirable	1-penten-3-ol, 3-methyl butanol, 2-octanone, 1-hexanol, acetic acid
Fruity	2-butanone, 3-methyl butanal, 2-methyl butyl propanoate, ethenyl benzene, 2-nonanone
Musty-humid	2-heptanone and 2-nonanonetrans
Metallic	1-penten-3-one
Rancid	unsaturated aldehydes

**Table 10:** Amount (%^a^) of bound phenolic compounds analyzed in full-fat and defatted olive cake extracts by using RP-HPLC [230].

Bound phenolic compounds	Full-fat olive cake^b^	Defatted olive cake^b^
Gallic acid	ND	ND
Protocatechuic acid	21.2 ± 0.24	13.8 ± 0.41
Hydroxybenzoic acid	5.8 ± 0.17	7.1 ± 0.17
Vanillic acid	4.9 ± 0.12	4.9 ± 0.43
Caffeic acid	13.7 ± 0.28	11.1 ± 0.25
Syringic acid	22.4 ± 0.38	22.7 ± 0.36
Sinapic acid	13.1 ± 0.29	16.6 ± 0.59
Ferulic acid	7.2 ± 0.08	7.9 ± 0.35
*p*-Coumaric acid	ND	ND
Rutin	11.7 ± 0.39	8.2 ± 0.24
Hesperidin	ND	4.3 ± 0.13
Quercetin	ND	3.4 ± 0.19
Cinnamic acid	ND	ND

ND: not determined.

a Mean value of three replicates ± standard deviation.

b Percentage of total phenolic content based on peak areas.

**Table 11:** Chemical composition of olive whole stones and olive seeds (as % dry weight) [242].

Components	Whole stone (%, w/w)	Seed (%, w/w)
Ash content	0.01–0.68	0.03–0.13
Moisture content	9.79	9.98
Fat	5.53	1.01
Protein	3.20	1.29
Free suger	0.48	0.36
Phenolics	0.1	0.5–1

**Table 12:** Effect of different cultivars on the phenolic compounds in olives.

Cultivar	Total phenols (mg/kg)	Reference
Arbequina	108.27	[54]
Arbosana	137.84	[54]
Koroneiki	236.48	[54]
Picual	400	[258]
Arbequina	334	[258]
Hojiblanca	355	[258]
Ornicabra	495	[258]
Leccino	130	[56]
Bianchera	305	[56]
Busa	125	[56]
Arbequina	243.8	[259]
Picolimon	159.9	[259]
Morisca	435.4	[259]

**Table 13:** Concentration of polyphenolic compounds recovered in the olive oils produced by using different crushing methods [286,288].

Compounds	Stone (ppm)	Hammer (ppm)
Gallic acid	1.60 ± 0.20	1.32 ± 0.14
Tyrosol	2.99 ± 0.17	3.00 ± 0.54
Vanilic acid	1.83 ± 0.12	1.27 ± 0.12
*p*-Coumaric acid	2.25 ± 0.16	1.97 ± 0.42
Ferulic acid	1.95 ± 0.12	1.62 ± 0.22
Luteolin	4.66 ± 0.25	4.20 ± 0.31
*trans*-Cinnamic acid	0.12 ± 0.01	0.11 ± 0.01
Apigenin	1.64 ± 0.17	1.61 ± 0.14

**Table 14:** Phenolic composition (ppm) of Coratina virgin olive oils with two phase and three phase centrifugation [295].

Phenolic composition	Two phases (ppm)	Three phases (ppm)
(3,4-DHPEA) Hydroxytyrosol	0.87 ± 0.02	0.58 ± 0.08
(*p*-HPEA) Tyrosol	3.74 ± 0.07	2.34 ± 0.08
Vanillic acid	0.41 ± 0.01	0.19 ± 0.01
Caffeic acid	0.16 ± 0.01	0.12 ± 0.02
(3,4-DHPEA-EDA) 3,4-dihydroxyphenyl-ethanol linked to elenolic acid	522.2 ± 13.5	427.2 ± 13.8
(*p*-HPEA-EDA *p*-hydroxyphenylethanol linked to dialdehydic form of elenolic acid	78.16 ± 0.52	67.26 ± 2.55
*p*-HPEA-ester	38.41 ± 0.10	35.62 ± 1.11
(3,4-DHPEA-EA)3,4-dihydroxyphenyl-ethanol linked to elenolic acid	351.71 ± 11.0	244.9 ± 13.6
Total polyphenols	673 ± 4	585 ± 7
